# 80 Years in Bath

**Published:** 1992-12

**Authors:** M. G. Wilson


**80 YEARS IN BATH**
**The Minutes of the Clinical Society of Bath 1908-1988**
Edited by Peter Henderson. Published by Addkey Print Ltd., The Precinct Church, Corsham, Wilts.

Dr. Peter Henderson, President of the Clinical Society of Bath in 1987 decided to produce an edited version of the minutes of the society from its inception in 1908. The result is a narrative that is surprisingly readable and records faithfully what the medical fraternity in Bath were thinking and writing papers about, the clinical cases they presented and how they conducted their affairs. The book which runs to 248 pages was obviously a labour of love for its compiler and will be of great interest to members of the Clinical Society of Bath. Leafing through its pages my interest was constantly aroused by expressions of current opinion on current practice as recorded in the discussions often enlivened with flashes of wit. Not all the entries record 'parish affairs'. Some of the speakers were of international eminence on subjects of cosmic inportance. Sir Fred Hoyle spoke to the Society in 1979 on his theory of the continuous creation of virus particle in outer space, how they cause episodes of influenza and why he thinks life must have started extraterrestially. It is the sort of archive from which the medical historian of the future will be able to learn how medicine progressed in the greater part of the twentieth century. It contains no index and that diminishes its usefulness to anyone wishing to research a specific interest, otherwise a model which other medical societies could well emulate.

M. G. Wilson

